# Treatment of Softened Dentin before Filling

**Published:** 1900-03-15

**Authors:** J. Leon Williams


### TREATMENT OF SOFTENED DENTIN BEFORE FILLING.

Remove the softer portions of dentin and place a pledget of cotton wool, saturated with absolute alcohol, in the cavity. Leave this for one minute, then remove, dry the cavity and flood it with oil of cloves, which also leave for one minute. Any one accustomed to histological work will see the *rationale* of this treatment at a glance. Oil of cloves, which is known to the histologist as one of the most powerful clearing agents known—*i. e.*, it has the property of very rapidly penetrating any tissue, even bone and dentin, that has previously been treated with strong alcohol. It is a sufficiently good germicide for the purpose, and it seems also to have mechanical effect of value in slight congestion of the pulp. Used as above described, it will penetrate a considerable thickness of dentin, and thus search out and destroy or render inert any forms of bacteria that may have penetrated beyond the point where you have cut. Dry out the excess of oil of cloves, and varnish the bottom of the cavity with Canada balsam, dissolved in chloroform, to which has been added 10 percent of the solution of hydronapthol in chloroform previously spoken of. For this use the balsam is dissolved in chloroform, instead of turpentine, because here we wish it to dry rapidly, while in the treatment of the root-canal we do not wish it to dry rapidly. Partially dry the layer of varnish in the bottom of the cavity with hot air, and then apply to the floor of the cavity a piece of thick asbestos paper cut the proper size and shape. The partially dried varnish will hold the asbestos paper firmly in place. Now line the cavity with quick-setting cement, and fill with gold or amalgam. Such treatment will leave the tooth reasonably free from sensitiveness to the thermal change, even when the pulp is nearly exposed.—*J. Leon Williams, International.*

